# Too hot to die? The effects of vegetation shading on past, present, and future activity budgets of two diurnal skinks from arid Australia

**DOI:** 10.1002/ece3.3238

**Published:** 2017-07-26

**Authors:** Annegret Grimm‐Seyfarth, Jean‐Baptiste Mihoub, Klaus Henle

**Affiliations:** ^1^ Department of Conservation Biology UFZ – Helmholtz Centre for Environmental Research Leipzig Germany; ^2^ University of Potsdam, Plant Ecology and Nature Conservation Potsdam Germany; ^3^ UPMC Université Paris 06, Muséum National d'Histoire Naturelle, CNRS, CESCO, UMR 7204 Sorbonne Universités Paris France

**Keywords:** activity predictions, behavioral thermoregulation, *Ctenotus regius*, extrapolating experimental data, *Morethia boulengeri*, operative temperature, operative thermal environment

## Abstract

Behavioral thermoregulation is an important mechanism allowing ectotherms to respond to thermal variations. Its efficiency might become imperative for securing activity budgets under future climate change. For diurnal lizards, thermal microhabitat variability appears to be of high importance, especially in hot deserts where vegetation is highly scattered and sensitive to climatic fluctuations. We investigated the effects of a shading gradient from vegetation on body temperatures and activity timing for two diurnal, terrestrial desert lizards, *Ctenotus regius,* and *Morethia boulengeri*, and analyzed their changes under past, present, and future climatic conditions. Both species’ body temperatures and activity timing strongly depended on the shading gradient provided by vegetation heterogeneity. At high temperatures, shaded locations provided cooling temperatures and increased diurnal activity. Conversely, bushes also buffered cold temperature by saving heat. According to future climate change scenarios, cooler microhabitats might become beneficial to warm‐adapted species, such as *C. regius,* by increasing the duration of daily activity. Contrarily, warmer microhabitats might become unsuitable for less warm‐adapted species such as *M. boulengeri* for which midsummers might result in a complete restriction of activity irrespective of vegetation. However, total annual activity would still increase provided that individuals would be able to shift their seasonal timing towards spring and autumn. Overall, we highlight the critical importance of thermoregulatory behavior to buffer temperatures and its dependence on vegetation heterogeneity. Whereas studies often neglect ecological processes when anticipating species’ responses to future climate change the strongest impact of a changing climate on terrestrial ectotherms in hot deserts is likely to be the loss of shaded microhabitats rather than the rise in temperature itself. We argue that conservation strategies aiming at addressing future climate changes should focus more on the cascading effects of vegetation rather than on shifts of species distributions predicted solely by climatic envelopes.

## 1 INTRODUCTION

The strong dependence of ectotherms on temperature is frequently assumed to be a key underlying process for modeling their response to climate warming, especially through its impact on activity budgets (Angilletta, 2009). However, those predictions sometimes result in contradictory findings. Caruso, Sears, Adams, and Lips (2014) predicted an increase in daily activity budgets in North American salamanders resulting in metabolic expenditure associated with body size reductions. Conversely, Sinervo et al. (2010) predicted a tremendous reduction in activity budgets, which could restrain metabolic functions and potentially causing up to 39% loss of lizard populations worldwide by 2080. So far, assessments of activity budgets have usually overlooked key factors, such as thermoregulation and microclimate variability (Gunderson & Leal, 2015; Kearney, 2013; Kearney, Shine, & Porter, 2009). Terrestrial ectotherms such as reptiles were recently found to offset a rather low thermal plasticity by active thermoregulation (Gunderson & Stillman, 2015). Thermoregulatory behavior is likely to be present in all reptiles and includes seeking for optimal thermal environments by basking, warming up on substrate, cooling down in the shade, and shuttling between thermally different microhabitats (Arribas, 2010, 2013; Bogert, 1949; Ortega & Pérez‐Mellado, 2016). At the individual level, thermoregulatory behavior that adjusts body temperature according to microhabitat conditions might be even more important for securing activity budgets than the environmental conditions on large spatial scales (Adolph & Porter, 1993; Sears & Angilletta, 2015). Moreover, thermoregulatory strategies including thermoregulation effort and accuracy were found to vary strongly between hot and cold environments (Vickers, Manicom, & Schwarzkopf, 2011).

Most studies investigating the sensitivity of reptiles to climate accounting for thermoregulation or microhabitat variation were conducted in (sub)tropical and temperate regions (e.g., Amo, López, & Martín, 2007; Arribas, 2010; Aubret & Shine, 2010; Huang & Pike, 2011; Huang, Porter, Tu, & Chiou, 2014; Logan, Huynh, Precious, & Calsbeek, 2013; Ortega & Pérez‐Mellado, 2016; Ryan et al., 2016). In contrast, only a few studies have focused on such adaptation mechanisms in hot deserts (Barrows, 2011; Jezkova et al., 2015; Porter, Mitchell, Beckman, & Dewitt, 1973). However, deserts in particular are predicted to be severely impacted by climate warming (Reisinger et al., 2014). While temperate and tropical regions are covered with dense forests or grasslands, desert vegetation is usually rare and scattered while covering only a minor proportion of the soil. Thus, the responses of reptiles to rising temperatures in temperate or tropical regions cannot simply be transferred to deserts (see also Clusella‐Trullas, Blackburn, & Chown, 2011). Unlike temperate or tropical regions, one of the most critical challenges for reptiles in hot deserts is to stay cool (Kearney et al., 2009). Consequently, desert reptiles have evolved different kinds of behavior, enabling them to offset the impacts of hot temperatures (Bartholomew, 1964). These different kinds of thermoregulatory behavior can buffer climatic variations to some extent (Angilletta, 2009). Their efficiency strongly depends on the availability of alternative microclimatic conditions such as shade provided by vegetation cover (Kearney, 2013; Kearney et al., 2009). To understand the available activity budgets of reptiles in such regions, it is imperative to compare the thermal conditions in the gradients of the available scattered vegetation.

In this study, we investigated the effects of vegetation on body temperatures and activity budgets of two skink species in an arid region of New South Wales, Australia, to determine the activity budgets from the past (1985 to now) to the future (until 2090) climatic conditions. In our approach, we combined different data sets by calibrating high‐resolution experimental data to longer but less accurate time series with different temporal resolutions to assess the species’ responses to climate change. We specifically aimed at disentangling the effects of bush sizes, the vegetation gradient, and occasional shading from isolated trees. Based on these findings, we investigated how activity budgets have changed over time and will change in the future to assess the potential effects of climate change on species with similar traits.

## 2 MATERIALS AND METHODS

### 2.1 Study site and study species

The study was conducted in Kinchega National Park, New South Wales, Australia (32°28′S, 142°20′E). Kinchega is situated at the eastern margin of Australia's arid zone and characterized by high summer temperatures and low but highly variable rainfall without seasonal patterns (Robertson, Short, & Wellard, 1987). Kinchega shows typical characteristics of a hot desert under climate change (rising temperatures and more extreme rainfall patterns). This region is projected to undergo major climate change in the future with a warming of up to 4–6°C by the end of the century (Reisinger et al., 2014).

**Figure 1:**
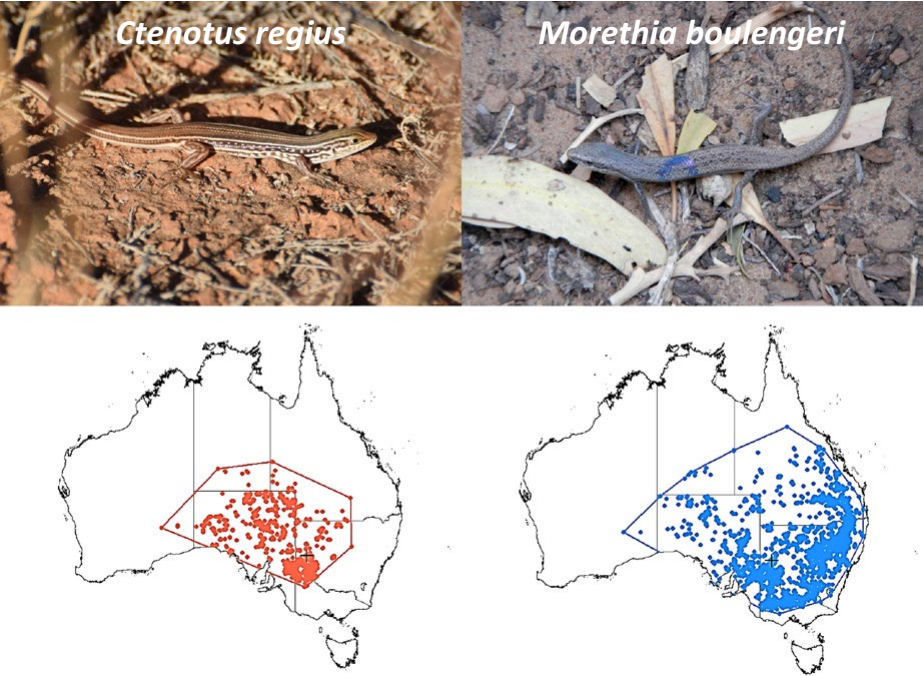
The terrestrial, diurnal skinks, *Ctenotus regius* (left), and *Morethia boulengeri* (right). The maps show the occurrence points and a minimum convex polygon of the species in Australia (data from: http://spatial.ala.org.au/). The black cross represents Kinchega National Park

Our study species are the terrestrial, diurnal skinks, *Ctenotus regius,* and *Morethia boulengeri* (Figure 1). While Kinchega's population of *C. regius* is located at the cold edge of the species distribution range, this geographic location represents the warm edge of the distribution range of *M. boulengeri* (Figure 1). Henle (1989a,b) found that repeated direct measurements of diurnal body temperatures of these lizards in this region are not feasible especially at hot temperatures, as individuals move too fast to be caught by hand and are too small be equipped with thermosensitive radio‐transmitters. Therefore, we used copper pipe models mimicking the bodies of lizards to measure the operative temperature of individuals *T*_e_ (i.e., the potential body temperature in a non‐thermoregulating individual). Copper pipe models are frequently used in field studies for the thermoregulation of small reptiles. They are assumed to have the same heat conductivity as an individual reptile (Bakken & Gates, 1975) and have been found to accurately predict steady‐state body temperatures of small individuals (Kearney et al., 2009; Seebacher & Shine, 2004). To ensure that our copper pipe models are a true mimic of individual's *T*_e_, we followed the suggestions made by Shine and Kearney (2001) and cut the copper pipes to the respective lengths and diameters of an average adult individual for each species (*C. regius*: 6.5 cm × 1.6 cm, *M. boulengeri*: 5 cm × 1.2 cm) and sealed both ends with polystyrene caps. To mimic the species’ reflectance, we dyed the copper pipes with a colored varnish in the respective colors of the species (*C. regius*: bright ivory (RAL 1015) with a black dorsal line, *M. boulengeri*: white aluminum (RAL 9006)) (Shine & Kearney, 2001). In each pipe, we placed an unwrapped and, in the case of *M. boulengeri*, sawn off iButton^®^ (DS1923) to log the temperature every 10 min.

Copper pipe models were placed at different locations of the habitat where each species is most common, respectively in red sand dunes dominated by Hopbush (*Dodonaea attenuata*) and blue bush *(Maireana pyramidata*) for *C. regius*, and in a riverine woodland of black box eucalypts (*Eucalyptus largiflorens*) and small bushes dominated by blue bush (*M. pyramidata*), *Sclerolaena paradoxa*, and *Enchylaena tomentosa* for *M. boulengeri* (Henle, 1989a,b, 1990). All models were loosely placed on the ground on similar soil types for each species and with a North‐South orientation for all measurements in order to minimize variations in *T*_e_ due to confounding factors (Shine & Kearney, 2001).

To ensure the representativeness of microhabitat conditions, we chose seven bushes of different sizes and measured *T*_e_ along the shading gradient of each bush between 3 February and 20 February 2015 and 9 February and 24 February 2016. This bush gradient comprised of five locations, starting outside of the bush (*T*_e.sun_) through three locations at the periphery (*T*_e.West_, *T*_e.East_, *T*_e.South_) to the bush center (*T*_e.shade_). We placed three copper pipe models per location per bush and used means across the three replications per location per bush for higher accuracy. For each T_e_ measurement, we recorded the bush species, the North–South and East–West extent, and the height of the bush. For measurements in the riverine woodland, we also noted whether the bush could be shaded by eucalypts. This approach is generally recommended for investigations of the spatial and temporal thermal structure of habitats (Vickers & Schwarzkopf, 2016).

### 2.2 Climate data

We used iButton^®^ temperature/humidity loggers (DS1923) to measure the environmental temperature in the air *T*_air_ (1.2 m above ground) and on the soil in direct sunlight *T*_sun_. For comparison, we measured the temperature in a burrow 15 cm below the surface *T*_burrow_ to evaluate whether individuals would survive in retreats. Environmental temperature measurements were conducted every 10 minutes at the same time as the measurements of *T*_e_ by the copper pipe models (February 2015 and February 2016). Additional environmental temperatures (*T*_air_ and *T*_sun_) were measured every three hours from February 2014 to February 2016. A detailed description of the climatic parameter space over these 2 years can be found in Appendix S1.

In order to model *T*_e_ in years where we did not measure environmental temperatures (see below), we obtained *T*_air_ from local weather data from the Bureau of Meteorology of the Australian Government (http://www.bom.gov.au/climate/data/stations) from the weather station closest to Kinchega (station 047019, Menindee Post Office). *T*_air_ was corrected according to the temperatures in Kinchega (Grimm et al. submitted).

### 2.3 Cooling power of bushes

We determined the cooling power of bushes at any time or day by calculating the maximum difference between *T*_e.sun_ and *T*_e_ measured at any other location below a bush, which resulted in 22,165 measurements. To investigate whether the size of the bush influenced its cooling power, we conducted a Pearson's correlation test between the cooling power and the bush size for each skink species.

### 2.4 From *T*_e_ to activity budgets

In order to extend the species‐specific *T*_e_ measurements to all those days where we had only measured environmental temperatures (February 2014–February 2016), we built a linear model (LM) using the copper pipe measurements of February 2015 and successfully validated the model using the copper pipe measurements from February 2016 (Appendix S2.1). We then extrapolated *T*_e_ from any other day and time between February 2014 and February 2016. Thus, we increased our overall time scale for which we could determine activity budgets from two month to two years, enabling more robust models for activity budget predictions in the past and the future (see below). Nevertheless, *T*_e_ values between February 2014 and February 2016 were on a coarser time scale (every 3 hours compared to every 10 minutes) and had to be calculated for three different bush types: no bush (i.e., *T*_e.sun_), small bushes, and large bushes. The size of the bushes emerged from average bush sizes measured in the field (*C. regius*: small = 4 m², large = 20 m²; *M. boulengeri*: small = 3 m², large = 10 m²). Moreover, *T*_e_ along the entire bush gradients, that is, through the locations at the periphery and bush center, was predicted.

We then calculated the corresponding activity budgets. We used two measurements for daily activity budgets: available activity time (AT) and relative available activity time (RelAT). We defined AT as the amount of time that an individual could be active within its operative thermal environment (Bakken, 1992). The operative thermal environment of a species reflects the thermal conditions of a specific location at a specific point in time within the species’ thermal activity range and at the appropriate time of the day (Porter et al., 1973). Following this definition, *C. regius* could be active at 19.3°C ≤ *T*_e_ ≤ 45.1°C from sunrise to sunset (Greer, 1989; Henle, 1989b), while *M. boulengeri* could be active at 12.7°C ≤ *T*_e_ ≤ 42.0°C from one hour before sunrise to one hour after sunset (Henle, 1989a,b). Data for sunrise and sunset were taken from Geoscience Australia, the Australian Government (http://www.ga.gov.au/geodesy/astro/sunrise.jsp).

For comparison, we defined daily RelAT as the percentage of AT in relation to the potential available time that a species would have on that day when ignoring thermal limits. Here, we did not differentiate between where individuals could be active, but rather between whether there was any location in the vegetation gradient where they could be active. In doing so, we assumed behavioral thermoregulation of individuals as they are assumed to shuttle between the most appropriate microhabitats.

### 2.5 Temporal extrapolation of relative available activity time (RelAT)

As we were interested in how RelAT changed from 1985 until now and how it might change until 2050 and 2090, we first had to predict RelAT for any other day that we had not measured. Therefore, we related species‐ and bush‐specific RelAT to the *T*_air_‐range of a given day between February 2014 and February 2016 (Appendix S2.2). Based on this relationship, we were able to predict RelAT on a daily basis for the last 30 years (1985–2016) using the available *T*_air_ time series and by cutting the values to the range of 0%–100%.

We then predicted *T*_air_ under climate warming according to the worst case IPCC emissions scenario RCP 8.5 in 2050 and 2090 (Appendix S2.2). These predictions resulted in a mean *T*_air.max_ of 30.10°C in 2050 and 32.51°C in 2090. We did not consider a more benign emissions scenario because our predictions of T_air.max_ were still below a continuation of the current linear trend in warming (32.42°C and 35.81°C in 2050 and 2090, respectively). We then used the predicted daily *T*_air.max_ and *T*_air.min_ values to predict the RelAT for every day of the year in 2050 and 2090.

To investigate whether RelAT changed over time, we finally used a linear mixed model (LMM) with RelAT as the response variable. The explanatory variables were the fixed effects of year and the quadratic relation of the Julian Calendar Day and the random intercept of year (Barr, Levy, Scheepers, & Tily, 2013). We conducted these LMMs separately for each species, bush type, and season (summer: October–March; winter: April–September) as we assumed different thermoregulatory behaviors between summer activity and winter activity (Appendix S1). Furthermore, we conducted these analyses twice – the first time to determine the past changes of RelAT (i.e., between 1985 and 2016) and the second time to include future changes of RelAT (i.e., between 1985 and 2090).

All statistical analyses were performed in R 3.1.1 (R Core Team 2016) unless otherwise stated. We used the packages *lme4* (Bates, Maechler, Bolker, & Walker, 2015) and *nlme* (Pinheiro, Bates, DebRoy, & Sarkar, 2016).

## 3 RESULTS

### 3.1 Operative temperatures and the cooling power of bushes

**Figure 2:**
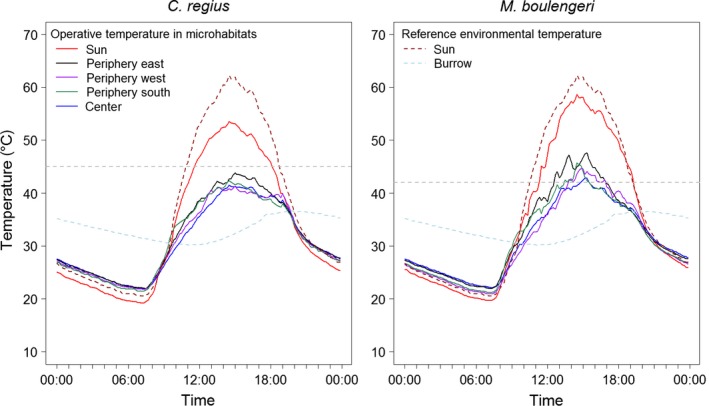
Average diurnal operative and environmental temperatures for *C. regius* (left) and *M. boulengeri* (right) at five different microhabitat locations across all days and bushes. The horizontal dashed gray line indicates the CTmax for each species

Despite daily fluctuations, operative temperatures *T*_e_ showed a consistent variation pattern within locations (Figure 2 for averages across all days). During the daytime, *T*_e.sun_ was much higher than *T*_e_ at any other location in the bush gradient for both species. *T*_e_ was almost always coolest in the bush center followed by *T*_e_ in the periphery with the warmest *T*_e_ always being the one under the sun's rays during daytime. At nighttime, no difference between bush locations was observed and *T*_e_ under any location of the bush gradient was slightly higher than *T*_e.sun_, that is, bushes were saving heat. Moreover, *T*_e.sun_ was lower than T_sun_ throughout the night. Generally, *T*_e_ followed *T*_sun_ (maximal range: 13.6°C–71.9°C) which was found to be stronger for *M. boulengeri* (maximal range: 13.5°C–68.7°C) than for *C. regius* (maximal range: 12.4°C–62.5°C). *T*_e.sun_ exceeded the species’ CTmax during the day (from 11:30 to 18:00 for *C. regius* and from 11:30 to 19:00 for *M. boulengeri*). However, bushes provided thermal refuges for individuals – although with species‐specific differences. All locations in the bush gradient kept the temperature below CTmax at any time of the day for *C. regius*. Contrastingly, only the bush center was found to be suitable throughout the day for *M. boulengeri,* while *T*_e_ in locations of the periphery was above CTmax between at least 14:00 and 15:00 on average (Figure 2). In comparison, we measured environmental temperatures in a burrow as a possible retreat site (*T*_burrow_) to investigate whether the species could survive at times of inactivity. We found that *T*_burrow_ was always well below the CTmax of both species. *T*_burrow_ showed a low total diurnal variation with a decreasing temperature until 13:00 (minimum value measured: 27.1°C) and an increasing temperature until 19:00 (maximum value measured: 39.1°C) (Figure 2).

**Figure 3:**
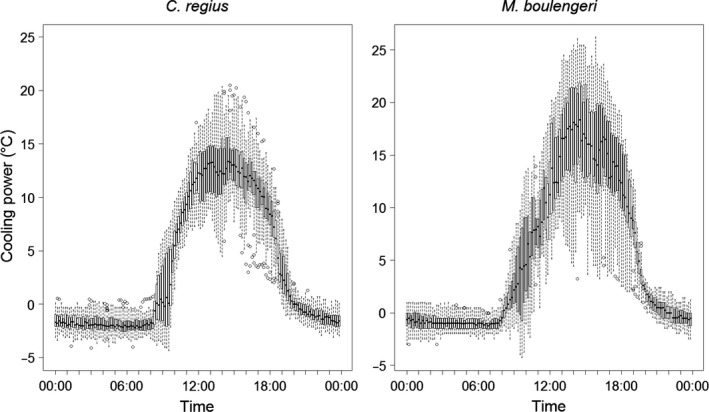
Average and range of the cooling power of bushes for *C. regius* (left) and *M. boulengeri* (right) throughout the day

Bushes acted as thermal regulators buffering external temperatures for both species either by cooling during the day or by saving the heat during the night. This regulation power depended on bush size. In general, larger bushes had a higher cooling power than smaller bushes (Pearson's correlation test: *t* = 9.48 and 11.98 for *C. regius* and *M. boulengeri*, respectively, *df* = 22163, *p* < 0.001). The variation between days and bushes was largest between 12:00 and 15:00 (Figure 3). Moreover, the cooling power for *M. boulengeri* was higher than for *C. regius* (difference to *T*_e.sun_ up to 26.2°C and 20.5°C, respectively) in spite of the biggest bush being 10.8 m² for *M. boulengeri* and 19.35 m² for *C. regius*. Additionally, the average *T*_e_ of *M. boulengeri* was 0.82°C lower if the area was occasionally shaded by a eucalypt tree (Appendix S2.1).

### 3.2 Available activity time under present conditions

**Figure 4:**
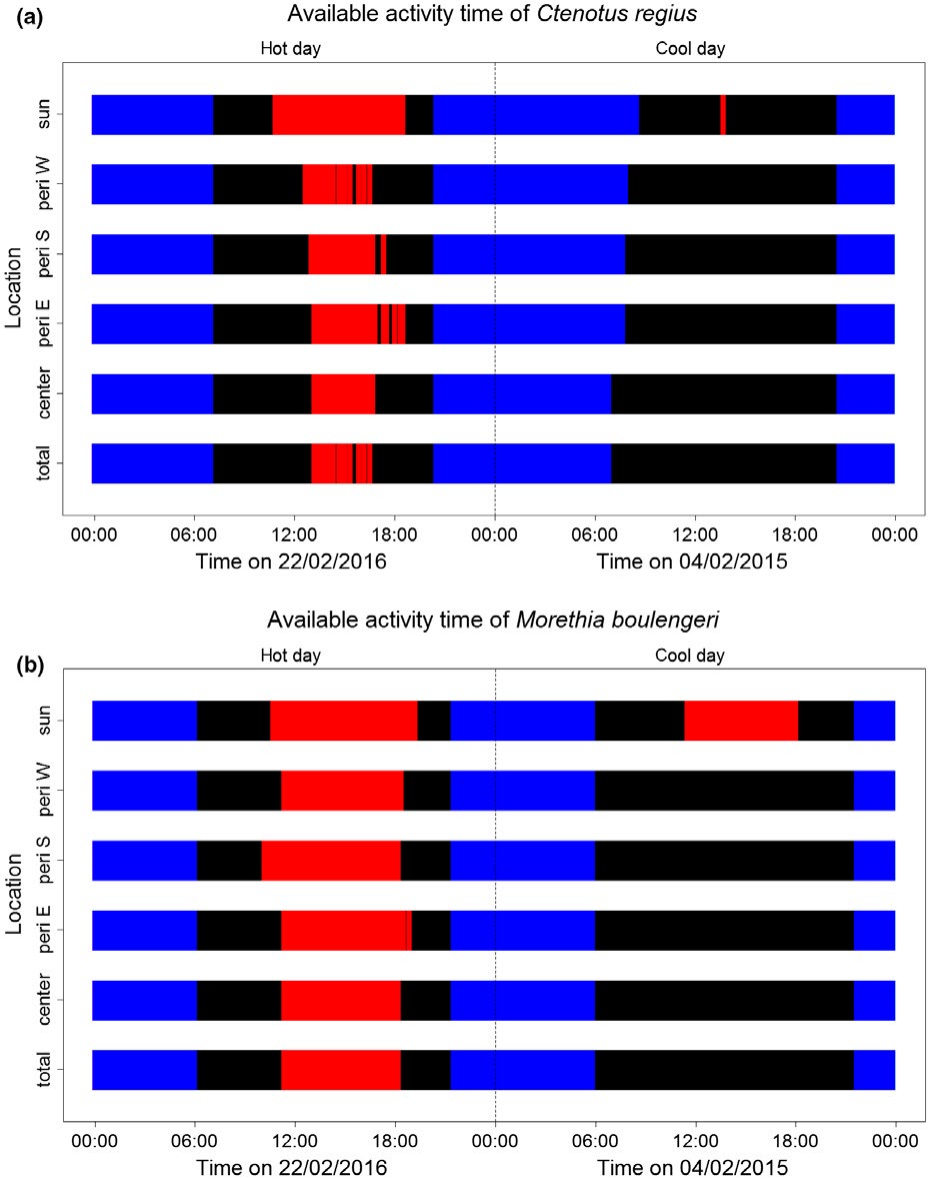
Comparison of available activity time on a very hot (22/2/2016) and a very cool (04/02/2015) summer day for *C. regius* (above) and *M. boulengeri* (below) across five locations. “Total” means the species can be active if it can be active at any location. Red areas symbolize inactivity due to temperatures above CTmax, blue areas symbolize inactivity due to night or temperatures below CTmin, and black areas symbolize activity

Heat reduced the duration of activity time (AT) in the sun for both species (Figure 4), both on hot days (e.g., 22/02/2016, *T*_air_ = 21.1°C–45.8°C, *T*_sun_ = 20.6°C–70.4°C; *C. regius* and *M. boulengeri* were restricted for 7.83 and 8.67 hr, respectively) and on cool days (e.g., 04/02/2015, *T*_air_ = 18.2°C–32.2°C, *T*_sun_ = 17.6°C–50.5°C; *C. regius* and *M. boulengeri* were restricted for 0.17 and 6.67 hr, respectively). In shaded locations of the bush gradient, daily AT restriction varied between locations on hot days, whereas both species were able to be active at all locations on cooler days (Figure 4). Overall, RelAT varied from 81% to 100% for *C. regius* with a mean of 95.3% (2015: 96.5%, 2016: 93.9%), and from 51.7% to 100% for *M. boulengeri* with a mean of 87.9% (2015: 92.6%, 2016: 82.8%). On the hottest days, total AT restriction (i.e., no activity at any location) was 2.5 and 7 hours for *C. regius* and *M. boulengeri*, respectively. In comparison, cold summer temperatures never restricted the AT of *M. boulengeri,* whereas the AT of *C. regius* was reduced due to the cold in the morning hours at all locations except for the bush center (Figure 4).

**Figure 5:**
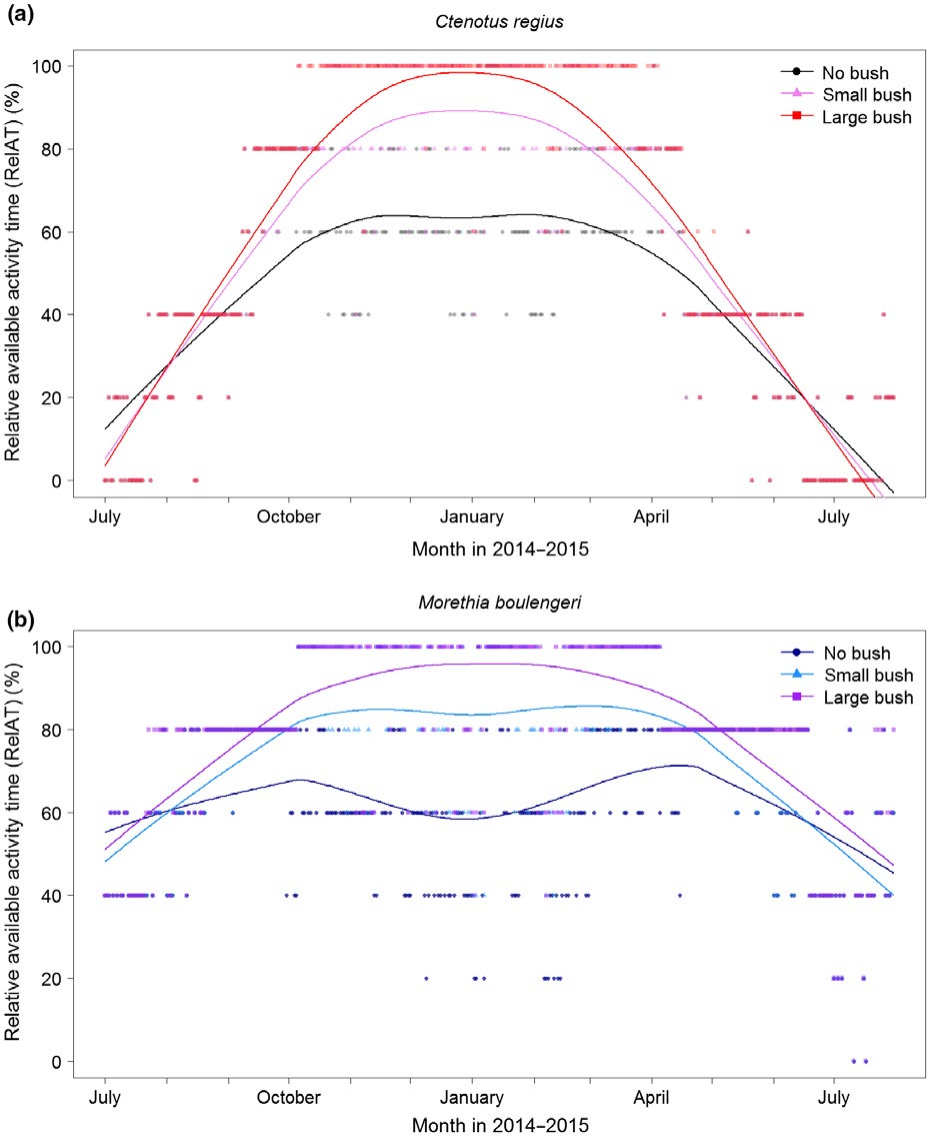
Relative available activity time (RelAT) preditions for *C. regius* (above) and *M. boulengeri* (below) across three bush types for one year. Symbols are calculated values, lines represent moving averages (smoother span factor 0.3)

When considered all year round (Figure 5), RelAT under large bushes was always higher than under small bushes or in the sun. In summer, both species were found to be fully active if large bushes were available (RelAT ≈ 100%), whereas they showed a restricted diurnal activity budget if only small bushes were available (RelAT = 80%–90%) or if no bush was present at all (RelAT = 60%–70%). The annual activity of *C. regius* still peaked in summer if only small bushes were available, but showed an annual bimodal activity without the presence of bushes. In comparison, *M. boulengeri* already showed an annual bimodal activity if only small bushes were available (Figure 5). In winter, the activity budgets of *C. regius* dropped to 20% or less irrespective of the presence of bushes with several days showing no activity at all. Winter activity budgets of *M. boulengeri* also dropped below 60%, but were above 20% all year round. Notably, its activity budget without the presence of bushes was identical between midwinter (July) and midsummer (January), implying strong restrictions due to the heat in summer and due to the cold in winter (Figure 5).

### 3.3 Available activity time under past and future conditions

**Figure 6:**
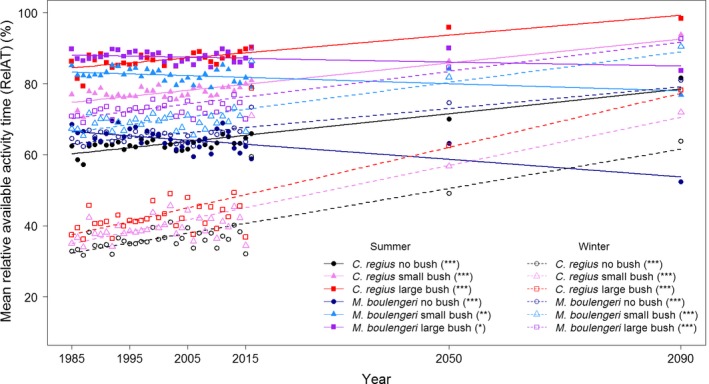
Mean relative available activity time (RelAT) across seasons for both skink species and three bush types. Symbols represent predicted values, lines are lines of best fit (filled and solid: summer, open and dashed: winter). Significance levels are shown in brackets in the legend

Activity budgets for *C. regius* were found to increase significantly for both time spans from the past to the present (0.003 ≤ *p* ≤ 0.007) and from the past to future conditions (*p* ≪ 0.001; Table 1; Figure 6) irrespective of the presence of bushes and for both seasons: summer and winter. On the contrary, activity budgets for *M. boulengeri* were found to decrease in the summer for both time spans from the past to the present (0.009 ≤ *p* ≤ 0.064) and from past to future conditions (0.001 ≤ *p* ≤ 0.047). They were only found to increase in the winter from past to recent conditions under large bushes (*p* = 0.033), but irrespective of bush presence from past to future conditions (*p* ≪ 0.001; Table 1; Figure 6). RelAT in winter always remained below the RelAT in summer for *C. regius* and increased by approximately 30% between 1985 and 2090 (Appendix S3, Figs S3.1, S3.3). On the contrary, the RelAT of *M. boulengeri* in winter was lower than in summer in the past. Nowadays, both winter and summer RelAT are almost equal but might be higher in the winter than in the summer by 2090, while still showing annual bimodal activity peaking in spring and autumn (Appendix S3, Figs S3.2, S3.3). Mean *T*_air.max_ and *T*_air.min_ as well as averaged RelAT separated by species, bush type, and season are summarized in Table S3.1 (Appendix S3).

**Table 1:** Estimates and *p* values for the fixed effect of year of an LMM investigating the temporal change of relative available activity time per day in Kinchega National Park

Time period			1985–2016		1985–2090	
Season	Bush type	Species	*Ctenotus regius*	*Morethia boulengeri*	*Ctenotus regius*	*Morethia boulengeri*
Summer	No	Estimate	0.077	−0.133	0.170	−0.116
		*p* value	0.007	0.009	<0.001	<0.001
	Small	Estimate	0.091	−0.074	0.165	−0.047
		*p* value	0.003	0.024	<0.001	0.004
	Large	Estimate	0.099	−0.050	0.135	−0.027
		*p* value	0.003	0.064	<0.001	0.047
Winter	No	Estimate	0.137	0.026	0.266	0.149
		*p* value	0.005	0.360	<0.001	<0.001
	Small	Estimate	0.176	0.072	0.322	0.207
		*p* value	0.007	0.078	<0.001	<0.001
	Large	Estimate	0.195	0.083	0.359	0.196
		*p* value	0.009	0.033	<0.001	<0.001

As the strongest differences in activity budgets between species occurred in summer, we will describe these changes in more detail (Appendix S3). In cooler years (i.e., in the 1980s and 1990s, Appendix S3 Table S3.1), *C. regius* showed an average daily activity budget of 80%–85%, while nowadays it has increased to 90% and is predicted to be above 95% in the future, when the presence of large bushes is assumed. Moreover, the number of days with RelAT of 100% in summer increased over time (Appendix S3, Figs S3.1, S3.3). Although *C. regius* always showed a unimodal annual activity distribution with one peak in summer, it might become bimodal in the future if no or only small bushes will be available (Appendix S3, Fig. S3.1). On the contrary, *M. boulengeri* always showed a slight bimodal activity distribution throughout the year with the strongest bimodality in areas without bushes (Appendix S3, Fig. S3.2). Activity budgets with up to 100% RelAT were found around April and November. When comparing past and future conditions, however, average summer activity decreased slightly from ≈90% and ≈80% for large and small bushes to ≈85% and ≈80% for now and ≈83% and 75% for the future, respectively (Appendix S3, Figs S3.2, S3.3).

We also found that the number of days within a year on which individuals could be active at least 80% of the day (Grimm et al. in prep.) was increasing for both species – irrespective of bush type (Appendix S3, Fig. S3.4). This increase continued until 2090 for *C. regius* but levelled off for *M. boulengeri* between 2050 and 2090 for areas with small or large bushes.

## 4 DISCUSSION

We found that the microhabitat (vegetation shading gradient and bush size) had a strong influence on the operative temperatures and the activity budgets of both species. At high temperatures, individuals of the two species benefited from shaded locations which decreased the operative temperatures and increased the activity budgets, especially for locations at the center of the bush and for large bushes. The shade of eucalypt trees additionally enhanced this cooling effect for *M. boulengeri*. However, bushes also enabled the two skink species to save heat when the temperature was cold with temperatures at central locations and large bushes cooling down more slowly than locations on the periphery of the bush gradient and smaller bushes. Thus, microhabitat conditions considerably increased the activity time for both species by balancing either warm or cold temperatures, confirming the critical influence of heterogeneous vegetation structures on both body temperature and activity (Kearney, 2013). Our results also showed fluctuations in the buffering effect of temperature along the vegetation gradient within the day which allowed behavioral thermoregulation in reptiles by providing alternative options while selecting optimal thermal places. Such active microhabitat selection behavior is important for the survival of lizards in hot deserts (Clusella‐Trullas et al., 2011; Vickers et al., 2011) and was frequently observed for both species in the field. Especially during summer, individuals were observed hunting in bushes and not in open areas in the early afternoon (Henle, 1989b; own observations).

In a detailed study of the reptile community of Kinchega National Park between 1985 and 1987, Henle (1989b) investigated the activity of both species through direct observations every second month. He found that *M. boulengeri* was active throughout the year with decreased activity in winter and hot summer months, while *C. regius* was only active from September to May with a peak of activity in hot summer months. Furthermore, he observed bimodal diurnal activity for *M. boulengeri* in November and January and for *C. regius* in January (there were no observations in December and February). Both observations are in line with our model predictions, strengthening the advantages of our approach that extrapolates fine‐scale experimental data to broadscale time series and that uses *T*_e_ to test whether the activity budget is an appropriate fit with the individual activity observed in the field.

The thermal preferences of the two species are also reflected by the location of Kinchega with respect to their distribution range: In our study area, the activity of *C. regius*, a species inhabiting Australia's hot central deserts (Figure 1), was most likely to be restricted by cold temperatures. Consequently, *T*_e_ in *C. regius* was certainly below CTmax most of the time, corroborating that hot periods enhance the activity time of this species in the study area. However, the restriction of activity on cold days even in summer or between autumn and spring was still very strong and *C. regius* had to shift its activity toward the warmer part of the day, that is, during early afternoon hours (Henle, 1989b; this study). Importantly, without large bushes saving heat in cold morning hours but cooling during hot early afternoon hours, *C. regius* was only able to be active on hot summer days until it became too hot and average summer activity would decrease by 20% per day (Appendix S3 Table S3.1). On the contrary, *M. boulengeri* inhabits cooler habitats toward Eastern Australia with Kinchega on the warm edge of its distribution area (Figure 1). Thus, *T*_e_ exceeded CTmax quite often, leading to frequent activity restrictions throughout summer months and bimodal activity. Although bimodal diurnal activity is a commonly observed behavioral strategy in desert lizards in summer (Adolph & Porter, 1993), the species’ activity in Kinchega would be restricted for an average of 40–50% of the day throughout almost the entire summer without the presence of large bushes under which they would be only restricted for an average of 10–15% per day (Appendix S3 Table S3.1). Our findings suggest that in addition to temperature as the most important driver (Cahill et al., 2014), the availability of vegetation and heterogeneity is highly important factors in determining the warm‐edge range limits for ectotherms. Likewise, Walker, Stuart‐Fox, and Kearney (2015) observed a warm‐edge range restriction in an Australian desert agama which was potentially driven by reduced midsummer activity budgets, not only depending on temperature but water and shelter availability. In addition, Clusella‐Trullas et al. (2011) found that precipitation rather than temperature is driving lizard performance, especially in arid areas. Although our study only examined the thermoregulatory options available to two lizard species, these two species represent the sympatric presence of warm‐adapted and cold‐adapted lizards in relation to the thermal habitat. Our predictions rely on thermal processes in ectotherms which do not differ fundamentally between species and regions. Differences would only occur if species were able to use a broader range of thermal habitats by either becoming nocturnal (Grimm, Prieto Ramírez, Moulherat, Reynaud, & Henle, 2014; Henle et al., 2010) or living in a subterranean environment where small changes in height can change the entire thermal conditions (Clusella‐Trullas et al., 2011; Henle, 1989c; Henle et al., 2010). As both species are diurnal and terrestrial, differences in their responses can only be explained by their adaptation to warm or cold habitats.

In the future, cooler microhabitats might become more favorable for *C. regius* which has already benefitted from a prolonged annual activity time, from which it might benefit even more in the future. In contrast, warmer microhabitat temperatures could well be above the thermal preferences of *M. boulengeri*, possibly restricting its activity completely during midsummers in the future. Hence, we could postulate that with climate warming warm‐adapted species might profit at their cold distribution edge, while cold‐adapted species might suffer at their warm distribution edge. Generally, climate warming will have less influence in shaded regions and a loss of shade in the future would be a more critical driver of reptile life histories and distributions than warming itself (Kearney, 2013; this study). As every type of vegetation might provide cooling effects (Huang et al., 2014; Kearney et al., 2009), future reptile distribution patterns would certainly be strongly affected by vegetation patterns (Sears et al., 2016). Modeling attempts to forecast future distributions of reptiles therefore critically need to consider more mechanistic processes to offer reliable and accurate predictions (Urban et al., 2016), for instance by integrating future vegetation patterns to reflect thermoregulation potential.

Thermoregulatory behavior might not shape the response to climate warming alone and could even limit a species’ potential for physiological adaptation (Buckley, Ehrenberger, & Angilletta, 2015). Instead of physiological adaptation, these species might shift their seasonal timing of activity. In line with that, we showed that with a warming climate, the total activity budget across the year was increasing for the two species investigated as it is the case for other desert species (Walker et al., 2015). However, the days of high activity budgets shifted to spring and autumn and the species might estivate in hot summer months in the future. As we showed that temperature in retreats (i.e., in burrows) was always below both species’ CTmax, estivation would not pose any risk of overheating at times of inactivity. In addition to seasonal shifts, Henle (1989b) observed a few individuals of *M. boulengeri* active at night suggesting some flexibility in the timing of activity in that species. Likewise, Treilibs, Pavey, Raghu, and Bull (2016) observed nocturnal activity of the desert skink *Liopholis slateri* during the hottest months. Nevertheless, it remains unclear how successful desert lizards would be able to change their diurnal or seasonal timing of activity. Generally, reptile species seem to have a large phenotypic plasticity, and an earlier spring and later fall provide a great opportunity for many species to increase their overall activity season (Bradshaw & Holzapfel, 2006, 2008; Walker et al., 2015) albeit this will depend on species‐specific genetic adaptation in photoperiodic responses (Bradshaw & Holzapfel, 2007). Comparably, widespread lizard species already show huge variability between phenological periods across latitudes with species with a shorter hibernation period often producing more and/or larger clutches (Grimm et al., 2014). In contrast, warmer hibernation periods in turtles led to greater energy losses during hibernation and in turn poorer body conditions during reproduction (Muir, Dishong, Lee, & Costanzo, 2013).

Together, not only the thermal preferences of the species but also the availability of vegetation and the seasonal timing of activity will determine whether a species can persist in a specific habitat (Kearney et al., 2009; Hacking, Abom, & Schwarzkopf, 2014; this study). While we cannot influence species’ adaptation mechanisms, we should preserve vegetation as refuges for small reptiles to increase the probability of persistence. In Australia, this means avoiding grazing and trampling by livestock or feral herbivores and preventing wildfires (Pavey et al., 2015) as well as preserving native vegetation and managing alien plants as their thermal microhabitats can differ substantially making alien plants unsuitable for small lizards (Hacking et al., 2014; Valentine, Roberts, & Schwarzkopf, 2007).

## 5 CONCLUSION

Extrapolating short term, high‐resolution experimental data to longer and less accurate time series is a promising approach to fill gaps in past records. Reconstructing past ecological conditions creates important challenges but is also imperative to address the long‐term responses of species to environmental changes. Here, we could stress that thermoregulatory behavior and the activity budgets of diurnal, terrestrial desert skinks were strongly impacted by the amount of vegetation and its heterogeneity, which provided both cooling spots and heat reservoirs. Although climate change is likely to lead to a species‐specific reduction in activity budgets in midsummer, it might also provide novel temporal niches that could even contribute to an increasing annual activity budget. Moreover, the cascading effects of vegetation rather than climatic envelopes alone should be addressed in future conservation strategies to prevent desert lizards from extinction.

## DATA ACCESSIBILITY

All abiotic data are available online: Climate data: Bureau of Meteorology, Australian Government (http://www.bom.gov.au/climate/data/stations). Sunrise and sunset data: Geoscience Australia, Australian Government (http://www.ga.gov.au/geodesy/astro/sunrise.jsp). Own measurements are available on Dryad: https://doi.org/10.5061/dryad.jg470.

## CONFLICT OF INTEREST

None declared.

## AUTHORS’ CONTRIBUTIONS

All authors conceived the ideas, designed the general methodology and collected field data. AG analyzed the data, performed the modeling and led the writing of the manuscript. All authors contributed critically to the drafts and gave their final approval for publication.
