# Supplementary material for: Too hot to die? The effects of vegetation shading on past, present, and future activity budgets of two diurnal skinks from arid Australia
Source: Ecol Evol. 2017 Jul 26;7(17):6803–13. doi: 10.1002/ece3.3238 (PMC5587462; doi:10.1002/ece3.3238)
Supplement: Supplementary file 1 [file ECE3-7-6803-s001.docx]

# Appendix S1: Climatic parameter space measured in 2014-2016

Temperature and humidity were measured every three hours from February 2014 to February 2016 using iButton® temperature/humidity loggers (DS1923). Maximum temperature was lowest in the air, followed by temperature on the soil surface in the shadow and temperature on the soil surface in the sun. While this difference was up to more than 30°C in summer, it almost vanished in winter (Figure S1.1, exemplarily for 2015). Minimum temperature was lowest in the air but almost identical between the soil surface temperature in the shadow and in the sun. There was no seasonal difference in this order (Figure S1.2, exemplarily for 2015).

The temperatures measured in the air were between -3.9°C and 48.6°C. Soil surface temperature in the shadow laid between 1.6°C and 63.4°C, while in the sun they rang was 0.12°C to 78.8°C.

Humidity varied between 0.2% and 100% in all locations. Temperature and humidity were strongly correlated across all locations (Pearson correlation test, p<<0.001). The hotter a day was, the less humid it was. Due to this strong correlation we excluded humidity as explanatory variable from all analyses.

**Figure S1.1.** Maximum daily temperature measured throughout the year 2015 in the air, on soil surface in the shadow and in the sun.

**Figure S1.2.** Minimum daily temperature measured throughout the year 2015 in the air, on soil surface in the shadow and in the sun.
