# Supplementary material for: Too hot to die? The effects of vegetation shading on past, present, and future activity budgets of two diurnal skinks from arid Australia
Source: Ecol Evol. 2017 Jul 26;7(17):6803–13. doi: 10.1002/ece3.3238 (PMC5587462; doi:10.1002/ece3.3238)
Supplement: Supplementary file 2 [file ECE3-7-6803-s002.docx]

# Appendix S2: Calibrating high-resolution experimental data to longer thermal time-series with different temporal resolutions

## S2.1: Extrapolation of T_e_ to environmental measures 2014-2016

In order to extend the species specific T_e_ measured by copper models in February 2015 and February 2016 to all days for which we only measured environmental temperatures (February 2014-February 2016), we build a linear model (LM) using the measures of February 2015 and validated the model using the measures from February 2016. After successful validation, we projected T_e_ at any other day and time between February 2014 and February 2016. In doing so, we increased our dataset for which we could determine activity budgets from two month to two years in order to get more robust models for activity budget predictions (see S2.2).

Specifically, T_e.sun_ (T_e_ in sun) was determined by an LM depending on soil surface temperature in the sun (T_sun_). At all other locations in the bush gradients, we used T_e_ as response variable and explained it with the LM

T_e_ ~ T_sun_*bush.area + T_sun_*TAVE_sun_*AMP_sun_ + location

with bush.area being the calculated bush area (N-S length ∙ E-W length) where T_e_ was measured, TAVE_sun_ being the temperature average of that day calculated as (T_sun.max_+T_sun.min_)/2, AMP_sun_ being the temperature amplitude of that day calculated as (T_sun.max_-T_sun.min_)/2, location being the location category where T_e_ was measured (periphery East, West, South, or centre of the bush), and * symbolising interaction. The interaction T_sun_*bush.area emerged from the fact that on a cool day the cooling power determined by the bush area was smaller and thus had less impact than on a hot day (see Appendix 1 and results in the main text). The relationship between T_e_ and T_sun_ further depended on the temperature behaviour throughout the day which is well described through TAVE and AMP (Reicosky *et al.* 1989). We assumed that the relationship of T_sun_ to TAVE and AMP reflected whether the bush was cooling or saving heat. For *M. boulengeri*, LM additionally contained a binary factor indicating whether the bush could be shaded by a eucalypt or not. We did not include the bush species as this was strongly correlated with bush.area (e.g., *D. attenuata* being larger than *M. pyramidata*).

As our data showed extremely strong temporal autocorrelation, which could not be solved by any autocorrelation term (results not shown), we randomly sampled half of the data to remove autocorrelation resulting in 4,718 data points. We ensured that this sampling procedure result in comparable sample size regarding the amount of data per day (270-321) and per location (923-960). After this sampling procedure, no deviation from any other model assumption could be detected. We performed two model validation steps. First, we checked how accurate the LM could predict T_e_ measurements in 2015 by checking the overall fit and deviance. For that we plotted the measured T_e_ in relation to T_sun_ and added the predicted T_e_ based on the LM separately for each skink species (Figure S2.1.1). We found that the predicted values mimic the variation in the measured values extremely well. Even extreme temperatures were accurately predicted.

Second, we predicted T_e_ based on the environmental data in February 2016 and checked whether predicted and measured T_e_ were comparable. For that we plotted predicted against measured T_e_ (Figure S2.1.2). We found that higher predicted values are in line with higher measured values. However, precision was higher for lower values of T_e_. Therefore, we compared the amount of T_e_ values above the species’ CTmax to ensure that we do not underestimate temperature extremes. For *C. regius*, we found that 614 measured and 700 predicted values of T_e_ were above the CTmax of 45°C. Likewise, for *M. boulengeri* 1345 measured and 1217 predicted values of T_e_ were above the CTmax of 42°C. As there was little difference between the measured and predicted values of T_e_ above CTmax, we expect that we cover extreme values well.

Parameter estimates and p-values can be found in Table S.1. We then used this LM to predict T_e_ according to the environmental temperatures (T_sun_) in 2014-2016. We predicted T_e_ at each location along the gradient in the bush assuming three different bush types: no bush (i.e., T_e_ in sun), small bushes, and large bushes. The size of the bushes emerged from average bush sizes measured in the field (*C. regius*: small = 4 m², large = 20 m²; *M. boulengeri*: small = 3 m², large = 10 m²).

Table S2.1: Parameter estimates and p-values of the LM to predict T_e_ at any location in the bush based on field measurements in 2015. The last line represents the results of the LM T_e.sun_ depending only on T_sun_.

| Test parameter | *C. regius* | | *M. boulengeri* | |
| --- | --- | --- | --- | --- |
|  | Estimate | p-value | Estimate | p-value |
| T_sun_ | 2.37 | <0.001 | 2.11 | <0.001 |
| bush.area | 0.47 | 0.031 | 1.30 | <0.001 |
| T_sun_ * bush.area | -0.01 | <0.001 | -0.03 | <0.001 |
| location | -0.60 – -0.52 | <0.001 | -1.10 – -0.39 | <0.001 |
| shade by Eucalypt | NA | NA | -0.82 | <0.001 |
| TAVE_sun_ | 1.71 | <0.001 | 1.49 | <0.001 |
| AMP_sun_ | 1.38 | <0.001 | 2.32 | 0.004 |
| TAVE_sun_ * AMP_sun_ | -0.06 | <0.001 | -0.07 | <0.001 |
| T_sun_ * TAVE_sun_ | -0.05 | <0.001 | -0.04 | <0.001 |
| T_sun_ * AMP_sun_ | -0.08 | <0.001 | -0.09 | <0.001 |
| T_sun_ * TAVE_sun_ * AMP_sun_ | 0.002 | <0.001 | 0.003 | <0.001 |
| T_sun_ | 0.83 | <0.001 | 0.90 | <0.001 |


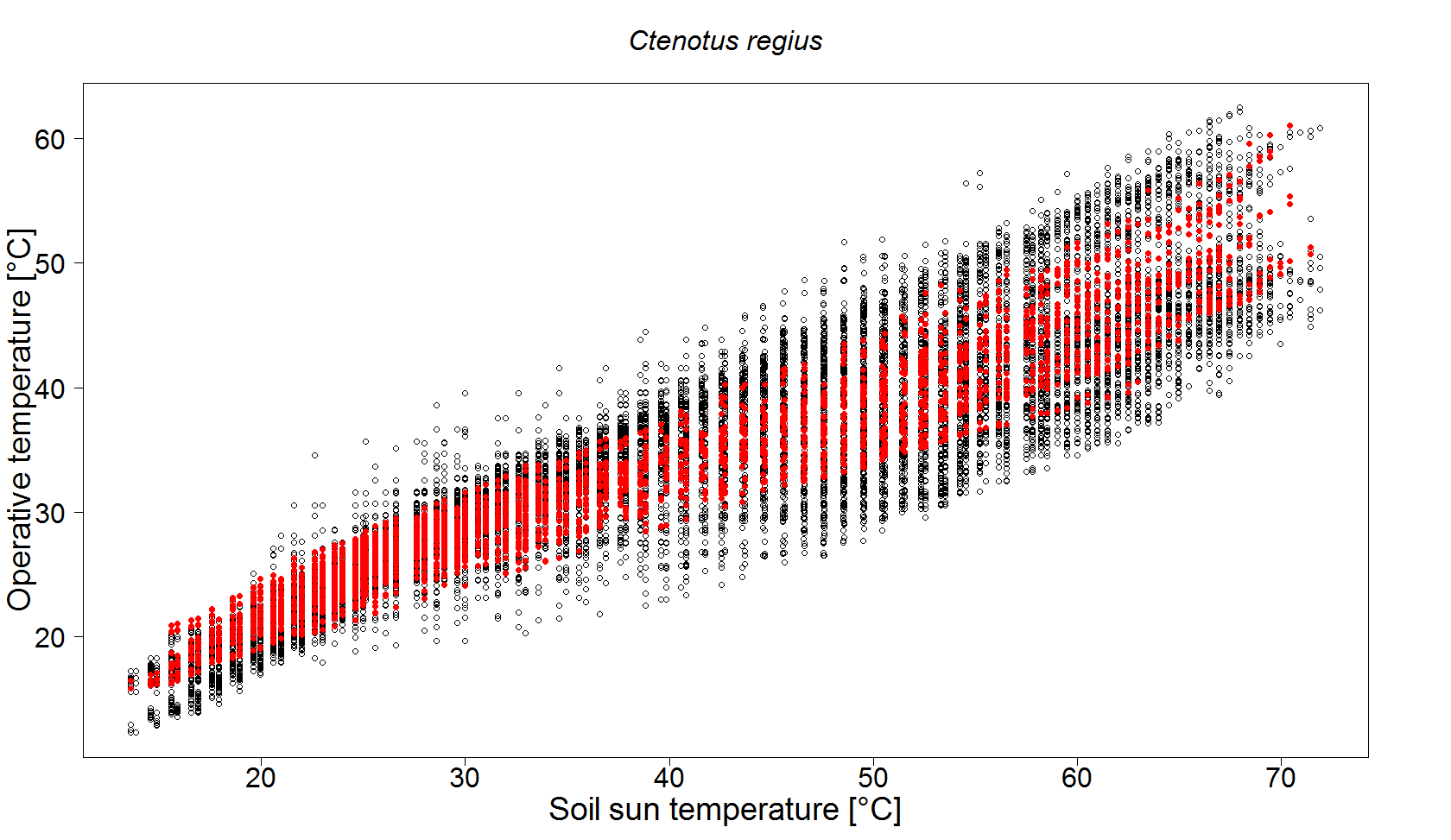


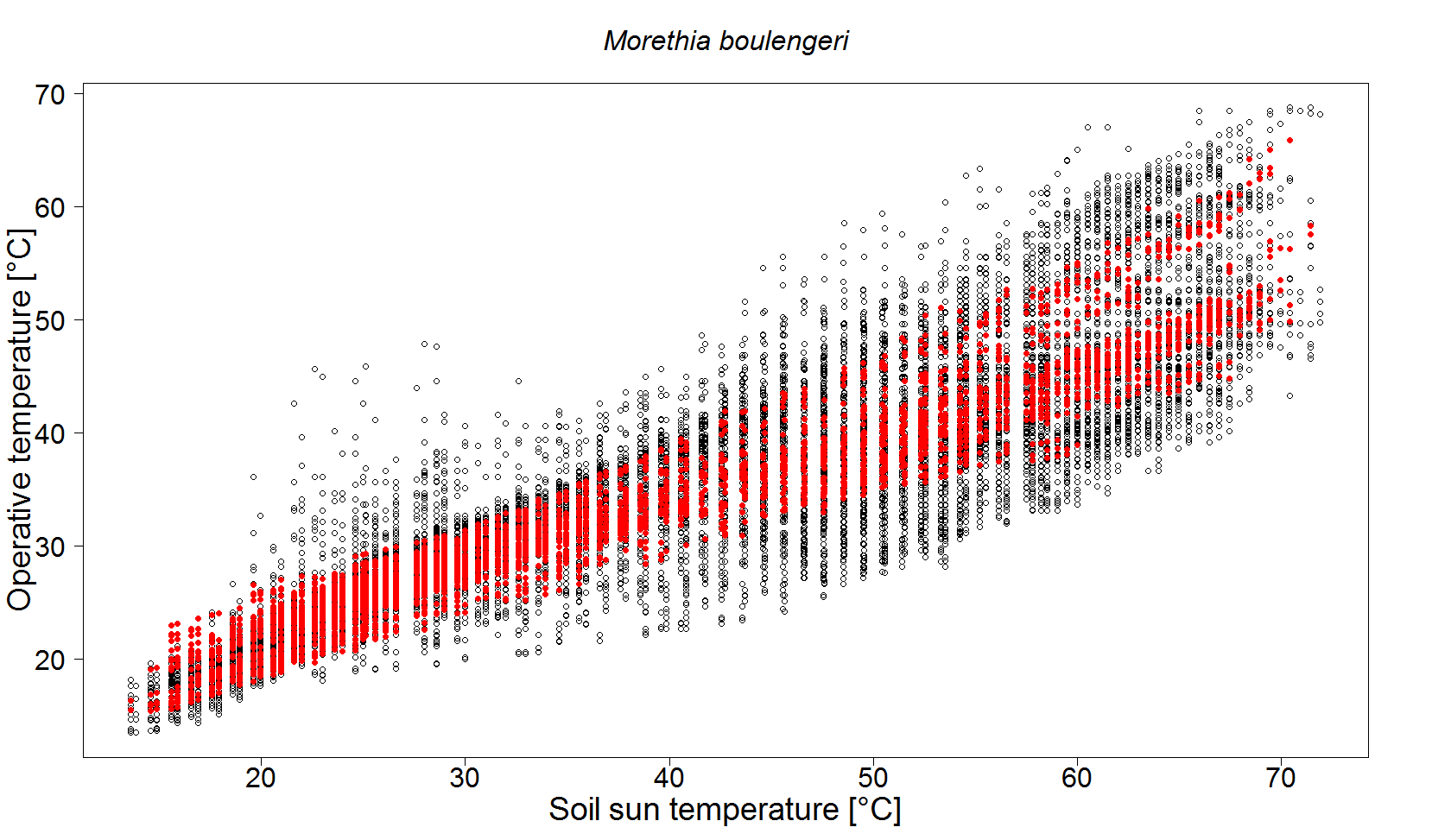


**Figure S2.1.1.** Operative temperatures T_e_ for *Ctenotus regius* (above) and *Morethia boulengeri* (below) in relation to soil surface temperature in the sun. Black dots represent measured values in 2015, red dots represent predicted values for 2015.


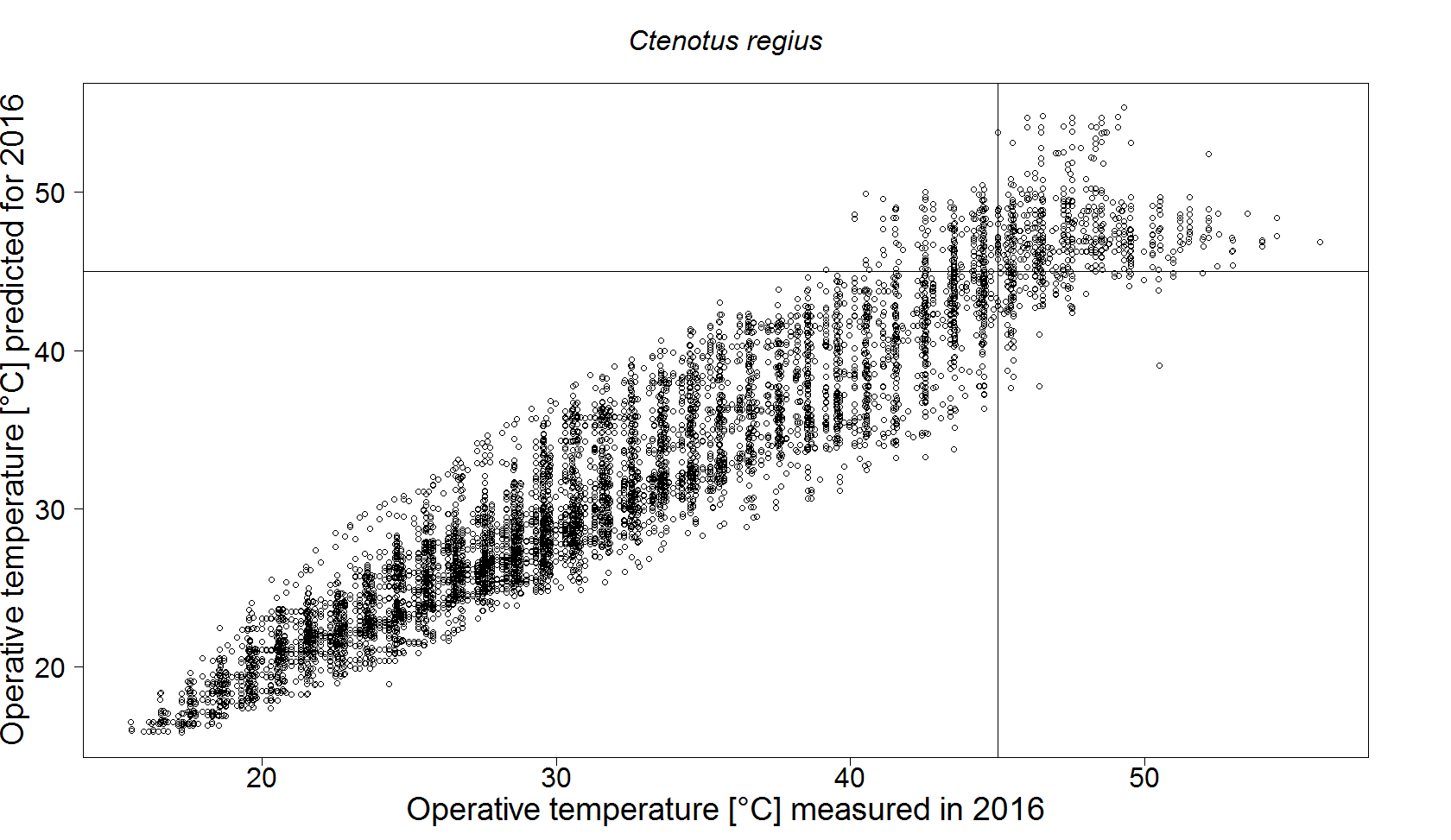


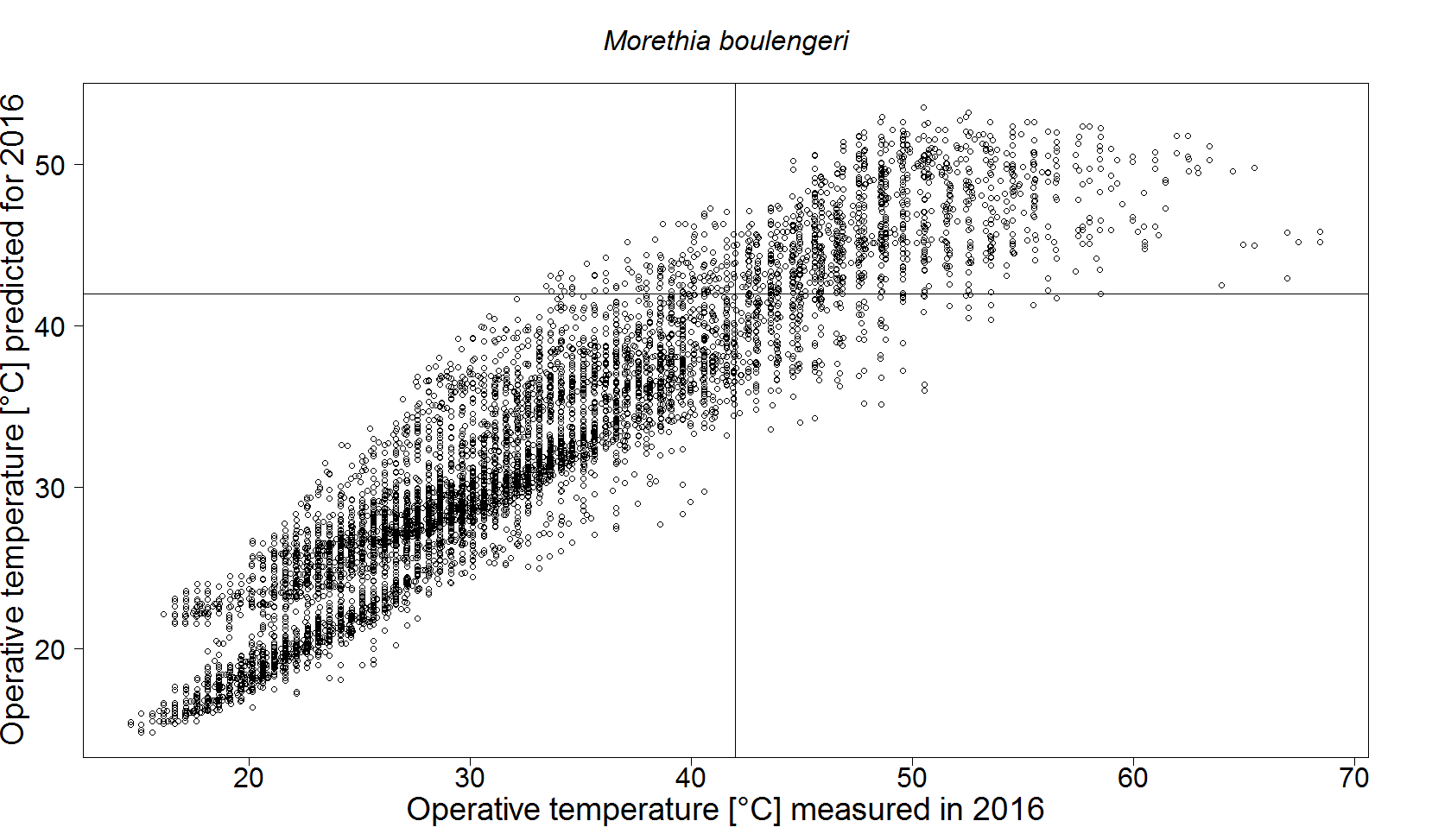


**Figure S2.1.2.** Predicted operative temperatures in relation to measured operative temperatures for *Ctenotus regius* (above) and *Morethia boulengeri* (below) in 2016.

## S2.2: Temporal extrapolation of relative available activity time (RelAT)

As we were interested in how the relative activity time (RelAT, see main text for calculation and definition) changed from 1985 until now and how it might change until 2050 and 2090, we first had to predict RelAT at any other day that we did not measure. The only environmental temperature available for decades and with at least a maximum and minimum value per day was air temperature (T_air_) (see Climate data section of the main text). Thus, we constructed an LM with RelAT per day (from February 2014 – February 2016) as response variable in relation to T_air_. We built separate LMs per species and per bush type (no bush, small, large). We did AIC comparisons of all model combinations with T_air.max_, T_air.min_, TAVE_air_, and AMP_air_ (Table S2.2) and found that the only model that always was among the most parsimonious models with the lowest AIC was

RelAT ~ TAVE_air_^2^* AMP_air_^2^

with TAVE and AMP being the average and the amplitude of air temperature a given day, respectively (Reicosky *et al.* 1989). These models were significantly different from the respective intercept models (p < 0.001) and did not show temporal autocorrelation or any deviation from model assumptions. This relation was then used to predict daily RelAT throughout the last 30 years and towards the future.

While maximum and minimum T_air_ values were available between 1985 and 2016, we had to predict them under climate warming assumptions for 2050 and 2090. We used the worst case IPCC emission scenario RCP 8.5. which predicts +2°C [+1°C; +3°C] for 2050 and +4.5°C [+3°C; +6°C] for 2090 relative to the temperature mean in 1986-2005 for this region (Reisinger *et al.* 2014). To predict the climate warming on a daily basis in 2050 and 2090, we simulated a set of daily temperature increases by creating a random normal distribution with 365 values per year with a mean ± standard deviation of 2 ± 0.5 and 4.5 ± 1, respectively, as the uncertainty is higher for 2090 (Reisinger *et al.* 2014). We then added these daily temperature increases to the daily T_air.max_ and T_air.min_ values averaged across 1986-2005. These predictions resulted in a mean T_air.max_ of 30.10°C in 2050 and 32.51°C in 2090. We did not consider a more benign emission scenario because our predictions of T_air.max_ were still below a continuation of the current linear trend in warming in Kinchega which would result in a mean T_air.max_ of 32.42°C and 35.81°C in 2050 and 2090, respectively.

Table S2.2: Model comparisons specific for each species and bush type. “+” indicate the explanatory variables used in an LM, “*” symbolises interaction with the previous parameter. The two models with the lowest AIC values are always highlighted.

| Species | bush type | T_air.max_ | T_air.min_ | TAVE_air_^2^ | AMP_air_^2^ | AIC |
| --- | --- | --- | --- | --- | --- | --- |
| *C. regius* | none | + | + |  |  | 6128.3 |
|  |  | + | * |  |  | 6123.1 |
|  |  |  |  | + |  | 6216.9 |
|  |  |  |  |  | + | 6714.0 |
|  |  | + |  | + |  | 6128.8 |
|  |  | + |  | * |  | 6111.5 |
|  |  |  |  | + | + | 6207.5 |
|  |  |  |  | + | * | 6112.0 |
|  | small | + |  |  |  | 6226.4 |
|  |  | + | + |  |  | 6138.2 |
|  |  | + | * |  |  | 6109.4 |
|  |  |  |  | + |  | 6205.6 |
|  |  |  |  |  | + | 6951.2 |
|  |  |  |  | + | + | 6188.8 |
|  |  |  |  | + | * | 6133.8 |
|  | large | + |  |  |  | 6152.0 |
|  |  | + | + |  |  | 6052.5 |
|  |  | + | * |  |  | 6000.7 |
|  |  |  |  | + |  | 6093.6 |
|  |  |  |  |  | + | 7053.0 |
|  |  |  |  | + | + | 6065.9 |
|  |  |  |  | + | * | 6030.6 |
| *M. boulengeri* | none | + |  |  |  | 5981.7 |
|  |  | + | + |  |  | 5927.9 |
|  |  | + | * |  |  | 5807.8 |
|  |  |  |  | + |  | 5958.5 |
|  |  |  |  |  | + | 6204.4 |
|  |  |  |  | + | + | 5885.2 |
|  |  |  |  | + | * | 5865.5 |
|  | small | + |  |  |  | 5978.5 |
|  |  | + | + |  |  | 5903.7 |
|  |  | + | * |  |  | 5831.1 |
|  |  |  |  | + |  | 5875.6 |
|  |  |  |  |  | + | 6307.7 |
|  |  |  |  | + | + | 5846.1 |
|  |  |  |  | + | * | 5845.9 |
|  | large | + |  |  |  | 5915.3 |
|  |  | + | + |  |  | 5859.6 |
|  |  | + | * |  |  | 5818.6 |
|  |  |  |  | + |  | 5839.0 |
|  |  |  |  |  | + | 6280.9 |
|  |  |  |  | + | + | 5825.3 |
|  |  |  |  | + | * | 5822.1 |

## References

Reicosky, D.C., Winkelman, L.J., Baker, J.M. & Baker, D.G. (1989) Accuracy of hourly air temperatures calculated from daily minima and maxima. *Agricultural and Forest Meteorology*, **46**, 193–209.

Reisinger, A., Kitching, R.L., F., C., Hughes, L., Newton, P.C.D., Schuster, S.S., Tait, A. & Whetton, P. (2014) Chap 25: Australasia. *Climate Change 2014: Impacts, Adaptation, and Vulnerability. Part B: Regional Aspects. Contribution of Working Group II to the Fifth Assessment Report of the Intergovernmental Panel on Climate Change* (eds V.R. Barros, C.B. Field, D.J. Dokken, M.D. Mastrandrea, K.J. Mach, T.E. Bilir, M. Chatterjee, K.L. Ebi, Y.O. Estrada, R.C. Genova, B. Girma, E.S. Kissel, A.N. Levy, S. MacCracken, P.R. Mastrandrea & L.L. White), pp. 1371–1438. Cambridge University Press, Cambridge, United Kingdom and New York, NY, USA.
