# Supplementary material for: Too hot to die? The effects of vegetation shading on past, present, and future activity budgets of two diurnal skinks from arid Australia
Source: Ecol Evol. 2017 Jul 26;7(17):6803–13. doi: 10.1002/ece3.3238 (PMC5587462; doi:10.1002/ece3.3238)
Supplement: Supplementary file 3 [file ECE3-7-6803-s003.docx]

# Appendix S3: Additional data and figures of relative available activity time throughout the past and the future

Table S3.1: Summary of mean annual air temperatures and averaged relative available activity time (RelAT) separated by species, bush type, and season.

| **Year** | **T_max_** | **T_min_** | **summer** | | | | | | **winter** | | | | | |
| --- | --- | --- | --- | --- | --- | --- | --- | --- | --- | --- | --- | --- | --- | --- |
|  |  |  | ***C. regius*** | | | ***M. boulengeri*** | | | ***C. regius*** | | | ***M. boulengeri*** | | |
|  |  |  | **none** | **small** | **large** | **none** | **small** | **large** | **none** | **small** | **large** | **none** | **small** | **large** |
| **1985** | 26.83 | 10.41 | 62.50 | 76.97 | 86.32 | 68.54 | 85.12 | 89.75 | 32.80 | 34.98 | 37.43 | 64.56 | 67.31 | 70.85 |
| **1986** | 26.78 | 9.49 | 58.61 | 72.37 | 81.38 | 66.21 | 82.38 | 87.03 | 33.30 | 36.60 | 39.44 | 63.44 | 67.18 | 70.94 |
| **1987** | 26.13 | 7.98 | 57.28 | 70.55 | 79.35 | 66.55 | 82.31 | 86.47 | 31.70 | 33.93 | 36.20 | 62.35 | 65.30 | 69.06 |
| **1988** | 28.89 | 11.46 | 62.47 | 77.85 | 88.02 | 63.05 | 81.98 | 87.02 | 38.24 | 42.31 | 45.73 | 67.01 | 71.69 | 75.15 |
| **1989** | 28.12 | 11.35 | 62.90 | 77.29 | 86.71 | 64.61 | 82.50 | 87.51 | 34.49 | 37.75 | 40.64 | 65.13 | 68.83 | 72.29 |
| **1990** | 28.06 | 12.33 | 63.60 | 78.78 | 88.47 | 63.81 | 82.29 | 87.58 | 34.22 | 37.50 | 40.68 | 66.40 | 70.04 | 72.88 |
| **1991** | 28.30 | 11.81 | 62.66 | 76.86 | 85.97 | 65.97 | 83.18 | 88.06 | 34.73 | 38.16 | 41.21 | 66.50 | 70.21 | 73.53 |
| **1992** | 26.26 | 11.02 | 62.87 | 76.11 | 84.51 | 69.60 | 85.14 | 89.72 | 32.04 | 34.10 | 36.40 | 63.85 | 66.50 | 69.88 |
| **1993** | 27.33 | 11.29 | 63.62 | 76.80 | 85.16 | 69.23 | 84.95 | 89.50 | 36.58 | 40.00 | 43.00 | 65.03 | 69.11 | 72.93 |
| **1994** | 28.13 | 10.12 | 61.52 | 76.24 | 85.81 | 65.09 | 82.55 | 87.73 | 35.77 | 37.73 | 39.87 | 63.71 | 66.77 | 71.41 |
| **1995** | 27.40 | 11.29 | 62.64 | 76.56 | 85.37 | 67.35 | 84.06 | 88.76 | 35.01 | 38.43 | 41.44 | 65.20 | 69.02 | 72.54 |
| **1996** | 27.06 | 11.09 | 62.19 | 76.39 | 85.56 | 67.87 | 84.44 | 89.21 | 34.92 | 38.15 | 41.15 | 66.54 | 69.95 | 73.19 |
| **1997** | 28.59 | 12.26 | 64.37 | 79.04 | 88.03 | 63.36 | 81.98 | 87.44 | 35.56 | 38.47 | 41.40 | 65.04 | 68.47 | 72.12 |
| **1998** | 27.64 | 11.86 | 63.52 | 77.97 | 87.26 | 66.76 | 84.04 | 88.95 | 35.47 | 38.79 | 41.91 | 66.33 | 70.03 | 73.37 |
| **1999** | 28.46 | 11.94 | 64.20 | 77.98 | 86.64 | 65.83 | 83.20 | 88.15 | 39.58 | 43.68 | 47.03 | 66.79 | 71.62 | 75.60 |
| **2000** | 27.78 | 11.76 | 65.32 | 79.64 | 88.67 | 65.79 | 83.65 | 88.74 | 36.27 | 39.40 | 42.26 | 65.75 | 69.45 | 73.10 |
| **2001** | 28.00 | 11.75 | 62.05 | 76.18 | 85.05 | 63.19 | 80.77 | 86.19 | 36.74 | 40.27 | 43.37 | 66.09 | 70.24 | 73.77 |
| **2002** | 29.34 | 11.45 | 62.91 | 77.70 | 87.20 | 64.32 | 82.46 | 87.65 | 41.10 | 45.58 | 49.05 | 65.55 | 71.19 | 75.69 |
| **2003** | 28.43 | 10.96 | 61.16 | 75.51 | 84.95 | 61.50 | 79.59 | 85.07 | 34.94 | 37.62 | 40.03 | 63.04 | 66.57 | 70.84 |
| **2004** | 28.91 | 10.72 | 61.24 | 76.55 | 86.62 | 62.18 | 80.76 | 86.24 | 36.42 | 39.37 | 42.03 | 63.44 | 67.65 | 71.93 |
| **2005** | 29.36 | 11.50 | 61.56 | 76.83 | 87.06 | 63.40 | 81.63 | 86.86 | 39.49 | 44.34 | 47.98 | 65.29 | 71.09 | 75.12 |
| **2006** | 29.38 | 11.42 | 63.51 | 78.85 | 88.64 | 59.52 | 79.50 | 85.34 | 33.73 | 35.62 | 37.61 | 62.53 | 65.48 | 69.96 |
| **2007** | 29.28 | 11.67 | 64.04 | 79.28 | 89.02 | 62.25 | 81.48 | 87.02 | 37.98 | 42.10 | 45.43 | 63.99 | 68.92 | 73.10 |
| **2008** | 28.56 | 10.88 | 62.79 | 77.68 | 87.39 | 64.62 | 82.70 | 87.91 | 35.93 | 38.44 | 40.69 | 63.55 | 67.16 | 71.61 |
| **2009** | 29.01 | 12.20 | 62.01 | 76.52 | 86.18 | 60.21 | 78.90 | 84.62 | 37.97 | 42.01 | 45.32 | 66.37 | 70.87 | 74.71 |
| **2010** | 27.03 | 11.97 | 63.33 | 76.76 | 85.43 | 67.37 | 83.87 | 88.59 | 33.70 | 36.39 | 39.22 | 65.76 | 68.89 | 71.97 |
| **2011** | 27.72 | 12.39 | 65.80 | 78.89 | 87.11 | 68.92 | 85.27 | 89.77 | 37.07 | 41.19 | 44.57 | 66.31 | 70.79 | 74.34 |
| **2012** | 27.93 | 11.84 | 65.03 | 79.10 | 87.82 | 66.58 | 83.99 | 88.94 | 36.24 | 39.60 | 42.51 | 65.16 | 69.24 | 72.89 |
| **2013** | 29.29 | 12.30 | 63.20 | 77.93 | 87.30 | 61.84 | 80.57 | 86.09 | 40.22 | 45.36 | 49.34 | 67.53 | 73.22 | 76.86 |
| **2014** | 29.36 | 12.33 | 63.21 | 78.89 | 89.03 | 60.46 | 80.20 | 86.01 | 38.34 | 42.26 | 45.53 | 66.53 | 70.91 | 74.67 |
| **2015** | 28.17 | 11.76 | 64.71 | 79.95 | 89.76 | 62.29 | 81.67 | 87.17 | 32.12 | 34.41 | 36.81 | 63.75 | 66.50 | 70.08 |
| **2016** | 34.22 | 17.28 | 65.95 | 80.85 | 90.35 | 58.87 | 79.08 | 85.08 | 59.42 | 71.02 | 78.78 | 73.39 | 86.34 | 90.12 |
| **2050** | 30.10 | 15.28 | 70.00 | 86.36 | 95.84 | 63.18 | 84.11 | 90.06 | 49.18 | 56.74 | 62.55 | 74.72 | 81.83 | 84.69 |
| **2090** | 32.51 | 20.19 | 81.68 | 93.82 | 98.37 | 52.42 | 76.80 | 83.68 | 63.81 | 71.91 | 78.19 | 80.99 | 90.45 | 92.65 |


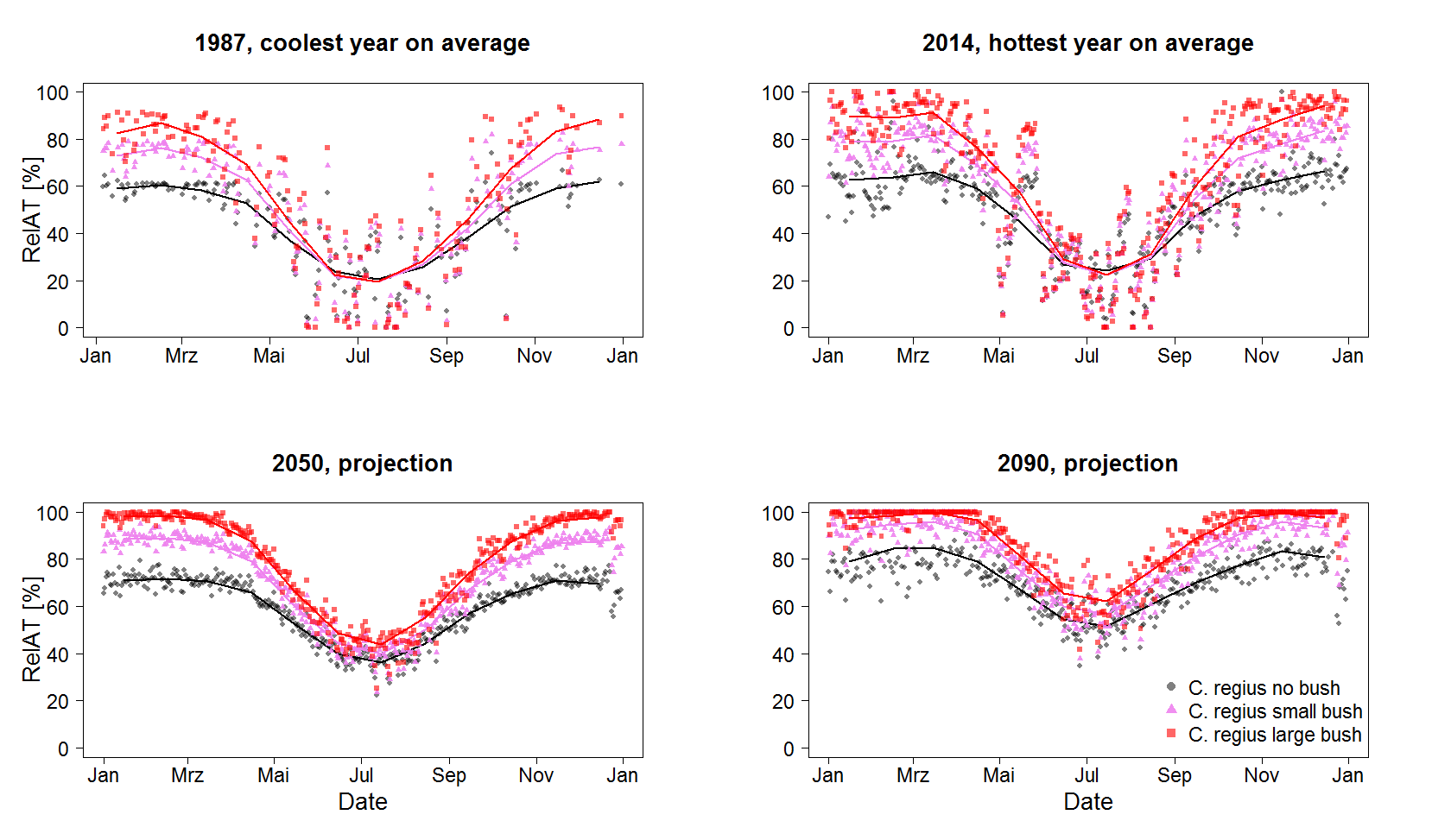


**Figure S3.1.** Relative available activity time (RelAT) in comparison between the coolest (1987) and hottest (2014) year in the past and the projected years in 2050 and 2090 for *C. regius*. Symbols represent daily RelAT, lines are connections between monthly mean values.


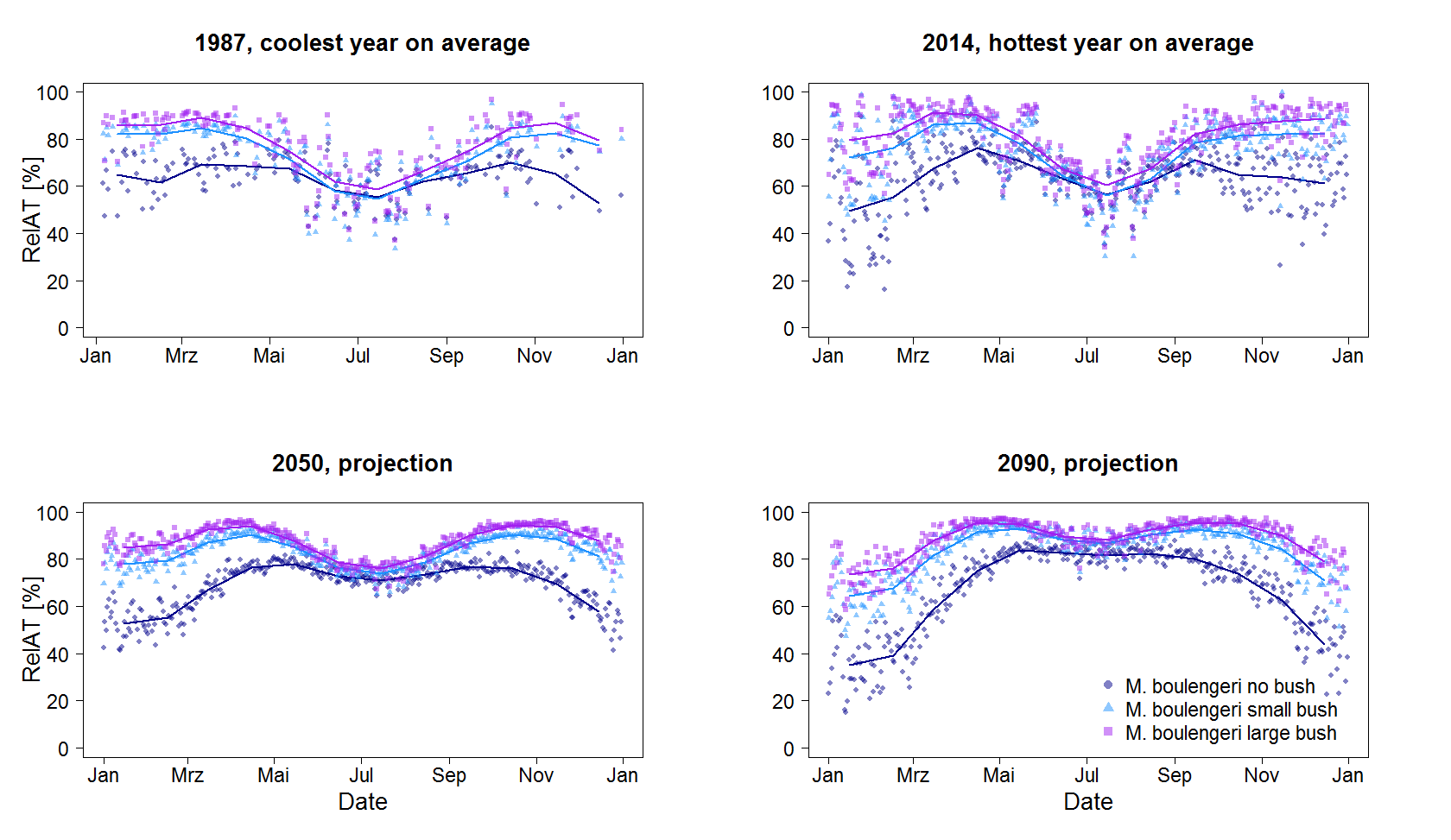


**Figure S3.2.** Relative available activity time (RelAT) in comparison between the coolest (1987) and hottest (2014) year in the past and the projected years in 2050 and 2090 for *M. boulengeri*. Symbols represent daily RelAT, lines are connections between monthly mean values.

**Figure S3.3.** Proportion per season spent in relative available activity time (RelAT) categories for *C. regius* (upper) and *M. boulengeri* (lower) in comparison between the coolest (1987) and hottest (2014) year in the past and the projected years in 2050 and 2090.

**Figure S3.4.** Total number of days above 80% relative available activity time (RelAT) per year for *C. regius* (left) and *M. boulengeri* (right). Symbols represent true values, dashed lines represent lines of best fit.
